# Brain Plasticity Modulator p75 Neurotrophin Receptor in Human Urine after Different Acute Brain Injuries—A Prospective Cohort Study

**DOI:** 10.3390/biomedicines12010112

**Published:** 2024-01-05

**Authors:** Santtu Hellström, Antti Sajanti, Abhinav Srinath, Carolyn Bennett, Romuald Girard, Ying Cao, Janek Frantzén, Fredrika Koskimäki, Johannes Falter, Seán B. Lyne, Tomi Rantamäki, Riikka Takala, Jussi P. Posti, Susanna Roine, Jukka Puolitaival, Miro Jänkälä, Sulo Kolehmainen, Melissa Rahi, Jaakko Rinne, Eero Castrén, Janne Koskimäki

**Affiliations:** 1 Department of Neurosurgery, Division of Clinical Neurosciences, Turku University Hospital, University of Turku, P.O. Box 52, Hämeentie 11, 20521 Turku, Finland; 2 Faculty of Medicine and Health Technology, Tampere University, 33520 Tampere, Finland; 3 Neurovascular Surgery Program, Section of Neurosurgery, The University of Chicago Medicine and Biological Sciences, 5841 S. Maryland, Chicago, IL 60637, USAcarolyn.bennett@bsd.uchicago.edu (C.B.);; 4 Department of Radiation Oncology, Kansas University Medical Center, Kansas City, KS 66160, USA; 5 Neurocenter, Acute Stroke Unit, Turku University Hospital, P.O. Box 52, 20521 Turku, Finland; 6 Department of Neurosurgery, University Medical Center of Regensburg, 93053 Regensburg, Germany; 7 Department of Neurosurgery, Brigham and Women’s Hospital, Harvard Medical School, Boston, MA 02115, USA; 8 Laboratory of Neurotherapeutics, Molecular and Integrative Biosciences Research Programme, Faculty of Biological and Environmental Sciences and Drug Research Program, 00100 Helsinki, Finland; 9 Division of Pharmacology and Pharmacotherapy, Faculty of Pharmacy, University of Helsinki, 00100 Helsinki, Finland; 10 Perioperative Services, Intensive Care and Pain Medicine, Turku University Hospital, University of Turku, P.O. Box 52, 20521 Turku, Finland; 11 Department of Neurosurgery, Oulu University Hospital, P.O. Box 25, 90029 Oulu, Finland; 12 Neuroscience Center, HiLIFE, University of Helsinki, P.O. Box 63, 00014 Helsinki, Finland

**Keywords:** neurotrophins, brain injury, stroke, trauma, outcome, recovery

## Abstract

Acute brain injuries (ABIs) pose a substantial global burden, demanding effective prognostic indicators for outcomes. This study explores the potential of urinary p75 neurotrophin receptor (p75NTR) concentration as a prognostic biomarker, particularly in relation to unfavorable outcomes. The study involved 46 ABI patients, comprising sub-cohorts of aneurysmal subarachnoid hemorrhage, ischemic stroke, and traumatic brain injury. Furthermore, we had four healthy controls. Samples were systematically collected from patients treated at the University Hospital of Turku between 2017 and 2019, at early (1.50 ± 0.70 days) and late (9.17 ± 3.40 days) post-admission time points. Urinary p75NTR levels, measured by ELISA and normalized to creatinine, were compared against patients’ outcomes using the modified Rankin Scale (mRS). Early urine samples showed no significant p75NTR concentration difference between favorable and unfavorable mRS groups. In contrast, late samples exhibited a statistically significant increase in p75NTR concentrations in the unfavorable group (*p* = 0.033), demonstrating good prognostic accuracy (AUC = 70.9%, 95% CI = 53–89%, *p* = 0.03). Assessment of p75NTR concentration changes over time revealed no significant variation in the favorable group (*p* = 0.992) but a significant increase in the unfavorable group (*p* = 0.009). Moreover, p75NTR concentration was significantly higher in ABI patients (mean ± SD 40.49 ± 28.83–65.85 ± 35.04 ng/mg) compared to healthy controls (mean ± SD 0.54 ± 0.44 ng/mg), irrespective of sampling time or outcome (*p* < 0.0001). In conclusion, late urinary p75NTR concentrations emerged as a potential prognostic biomarker for ABIs, showing increased levels associated with unfavorable outcomes regardless of the specific type of brain injury. While early samples exhibited no significant differences, the observed late increases emphasize the time-dependent nature of this potential biomarker. Further validation in larger patient cohorts is crucial, highlighting the need for additional research to establish p75NTR as a reliable prognostic biomarker across various ABIs. Additionally, its potential role as a diagnostic biomarker warrants exploration.

## 1. Introduction

Acute brain injuries (ABIs) such as aneurysmal subarachnoid hemorrhage (aSAH), ischemic stroke (IS), and traumatic brain injury (TBI) are considerable causes of mortality and disability worldwide [1,2]. Regardless of the underlying etiology, ABIs cause remarkable anatomical and functional changes to the brain, leading to temporary and/or permanent neurological deficits [3,4,5]. Treatment strategies for these conditions have advanced significantly, yet the clinical outcomes are still widely variable, making the prediction of outcomes a challenging task [6,7,8,9,10].

After the initial brain insult, complex biochemical and cellular processes are activated in the central nervous system (CNS) leading to dysfunction and apoptosis of neurons and supporting cells. These dysregulated processes and pathways offer targets for rehabilitation interventions [11]. Neuroplasticity plays a key role in neuronal rehabilitation inducing structural and functional nervous system reorganization in response to stimuli [12,13,14]. Recently, there has been an increased interest in understanding regarding the activity of biomolecules and their associated receptors that may play a role in neuronal plasticity and rehabilitation [15]. One such influential receptor is the p75 neurotrophin receptor (p75NTR), a member of the tumor necrosis receptor superfamily that functions as a pan-neurotrophin receptor. It engages a wide spectrum of ligands, spanning from proneurotrophins to fully matured neurotrophins such as nerve growth factor (NGF) and brain-derived neurotrophic factor (BDNF). Ligand interaction with p75NTR triggers signaling pathways that orchestrate neuronal processes encompassing survival, growth, differentiation, synaptic plasticity, apoptosis, and inflammation [15,16]. Neurotrophin signaling pathways generally involve two types of receptors: the Trk receptor tyrosine kinases (including TrkA, TrkB, and TrkC) and the p75NTR [15,16]. Each neurotrophin has a specific affinity for different Trk receptors (NGF for TrkA, BDNF and NT-4 for TrkB, and NT-3 for TrkC) and neurotrophins can also bind to p75NTR [16]. The interaction of neurotrophins with Trk receptors typically promotes cell survival and differentiation [16]. Upon binding to their respective Trk receptors, neurotrophins induce receptor dimerization and autophosphorylation, leading to the activation of intracellular signaling cascades. These cascades involve various pathways such as the MAP kinase, PI3 kinase, and PLCγ pathways, which collectively contribute to neuronal survival, growth, and synaptic strengthening [16,17]. When p75NTR interacts in complex with tropomyosin-regulated kinase (Trk) receptors, it increases its affinity for mature neurotrophins which initiates neuronal processes known to be associated with neuroprotective phenotypes such as cell survival, cell growth, and synaptic plasticity [15,17]. In contrast, proneurotrophin-mediated signaling pathways commonly induce adverse effects, such as apoptosis, neuronal cell death, and neurodegeneration [15,18]. It has been observed that the affinity of p75NTR for proneurotrophins increases when it forms a complex with sortilin, a Vps10p domain receptor [18,19,20]. Therefore, the current understanding of p75NTR suggests that it may act adversely or in a protective fashion after ABI depending upon which pathways are activated.

Although neurotrophic factors such as BDNF, NT3, and their respective receptors Trks are recognized in acute brain injury, our study specifically centers on the p75 neurotrophin receptor (p75NTR), given its unique role in various pathological neurological diseases and CNS injuries and especially the previous evidence to its potential role in prognostication from urine samples [21,22,23,24,25,26,27]. The overexpression of p75NTR has been observed in various studies within affected regions of the nervous system, contributing to the progression of neurodegenerative diseases and the exacerbation of secondary injuries following brain trauma [21,22,23,24,25,26]. Both in vitro and in vivo studies have demonstrated that pharmacological inhibition of p75NTR leads to significantly reduced pathological changes in various neurological conditions such as Alzheimer’s disease, Parkinson’s disease, and amyotrophic lateral sclerosis (ALS) [21,22,23,24,25,26,27]. Preclinical animal studies of brain injuries have demonstrated how various p75NTR inhibitors, including EVT901 and LM11A-31, induce neuroprotective effects following injury, suggesting that p75NTR may be a viable therapeutic target for future pharmacological investigations [25,28]. Recently, the first phase 2a clinical trials investigating the effects of LM11A-31 on patients diagnosed with Alzheimer’s disease were executed, but the results are not yet available (Clinical Trial Number^i^: NCT03069014) [29]. Additionally, recent animal model studies have observed that the administration of LM11A-31 improves the outcome in stroke model mice by decreasing neuronal degeneration, increasing levels of neurotransmitters (e.g., serotonin, acetylcholine, and dopamine), thereby attenuating motor function impairments [30,31]. Significant reductions in tissue damage and neurological deficits have also been achieved through this treatment through reduced proNGF activity, leading to a decrease in the apoptotic role of p75NTR and an increase in neuroprotective effects [31].

Furthermore, previous research by Jia et al. and Shepheard et al. from 2017 established that the extracellular domain of p75NTR (p75NTR^ECD^) was consistently higher in the urine of ALS patients than in healthy controls and seemed to increase as the disease progressed [32,33]. Thus, urinary p75NTR^ECD^ seems to have potential value as a prognostic biomarker for the disease [32,34]. Despite the recognition of p75NTR in neurodegenerative conditions, its potential as a urinary biomarker for prognosticating outcomes in various brain injuries remains largely unexplored. There is a lack of investigation into the prognostic value of p75NTR levels in urine for ABI outcomes. The need for non-invasive, easily accessible biomarkers in this domain would be valuable, given the complexities and limitations associated with current diagnostic modalities. In this study, we delve into the temporal concentration changes of p75NTR in urine samples, driven by the hypothesis that its increased levels could correlate with unfavorable outcomes after ABIs. By examining p75NTR in this light, our research aims to contribute to a deeper understanding of its prognostic and diagnostics capabilities.

## 2. Materials and Methods

### 2.1. Study Methods and Participants

**Figure 1:**
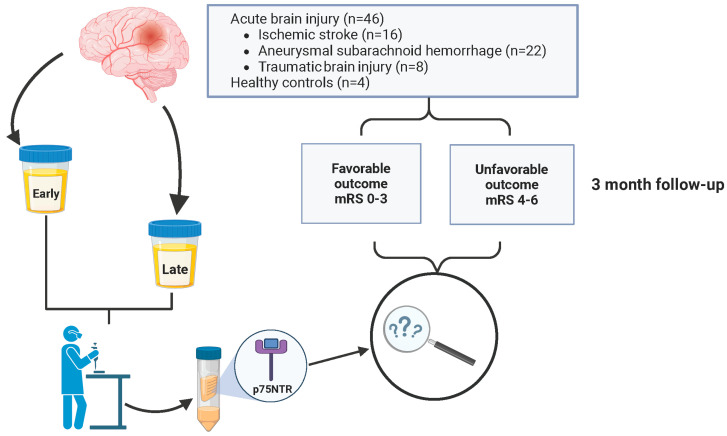
Flow chart of study design. The prospective cohort (*n* = 46) consisted of consecutively collected patients with ischemic stroke (IS) (*n* = 16), aneurysmal subarachnoid hemorrhage (aSAH) (*n* = 22), and traumatic brain injury (TBI) (*n* = 8). Patients were categorized based on their modified Rankin Scale (mRS) scores into favorable (mRS = 0–3) and unfavorable (mRS = 4–6) outcome groups. Urine samples were collected at two time points, early (1.50 ± 0.70 days) and late (9.17 ± 3.40 days), post admission. The concentration of p75 neurotrophin receptor (p75NTR) was measured from urine samples and adjusted for urine creatinine concentration.

We conducted a prospective cohort study in which urine samples from patients with an acute brain injury (ABI) were collected from University Hospital of Turku, Finland, where all the patients were admitted and treated between 2017 and 2019. Inclusion criteria were aneurysmal subarachnoid hemorrhage, ischemic stroke (thrombotic, embolic, or cryptogenic) or traumatic brain injury causing subdural hematoma requiring surgical evacuation. Patients were required to be over 18 years old, and informed consent was required. ABI patients were treated according to in-house protocols based on current recommendations of specific types of brain injury [35,36,37]. Urine samples were collected early (1.50 ± 0.70 days) and late (9.17 ± 3.40 days) after the insult. The cohort (*n* = 46) consisted of three groups: the aSAH group (*n* = 22), the IS group (*n* = 16), and the TBI group (*n* = 8). In addition, we included four (*n* = 4) healthy control patients with no neurological diseases. The patients were further divided into two outcome grades based on the modified Rankin Scale (mRS). Patients with mRS 0–3 were categorized as favorable, and mRS 4–6 were unfavorable. A structured outcome evaluation was performed after a three-month follow-up. Patients with aSAH came to an out-patient clinic, and IS and TBI patients were evaluated with a structured telephone interview. No study patients were lost to follow-up over the 3 months (Figure 1).

### 2.2. Study Approval and Ethics

The study was approved by the Turku University Hospital institutional review board and ethics committee (T291/2016) and was conducted according to the declaration of Helsinki and its later amendments. All participants provided written informed consent to undertake this current study. If it was not possible to obtain consent directly from the patient due to a severe acute illness, a legal trustee authorized it. The ethical principles guiding the institutional review board are consistent with the Belmont Report and comply with the rules and regulations of the legislation in Finland.

### 2.3. Assessment of Biomarkers

Urine samples were collected and stored according to the Urine and Kidney Proteome Project Standards. Samples were pulled in a sterile syringe from the catheter, centrifuged at 1000× *g* for 10 min to remove cells and debris, and 10 mL aliquots were stored at −80 °C. Samples were given deidentified codes. p75NTR concentrations of the urine samples were measured by using commercially available enzyme-linked immunosorbent assays (Invitrogen^®^, Waltham, MA, USA, Catalog number: EHNGFR). Furthermore, urinary creatinine measurements were performed using a Creatinine Urinary Detection Kit (Invitrogen^®^, Waltham, MA, USA, Catalog number: EIACUN).

Samples were loaded in parallel duplicate wells, with measurements obtained following the manufacturer’s instructions. Absorbance values were read at 450 nm using a 96-well microplate reader. For all ELISA tests, samples and standards were measured in duplicate, and the means of the duplicates were used for statistical analyses. One plate per disease group was used in both biomarkers; thus, a total of six ELISAs were performed (three p75NTR plates + three creatinine plates). The prevailing approach in the literature has been to investigate the association between p75NTR and creatinine (Crea) concentrations (ng/mg) because urinary protein biomarkers as a protein-to-creatinine ratio helps to mitigate variability and improve the accuracy, consistency, and clinical relevance of the findings. In this study, when referring to p75NTR concentration, we specifically denote the ratio of p75NTR (ng) to Crea (mg).

### 2.4. Statistical Analysis

Urine concentrations of p75NTR were standardized using urine creatinine levels, resulting in a p75NTR/creatinine ratio. To compare the differences in this ratio between patients with favorable outcomes (mRS scores of 0–3) and those with non-favorable outcomes (mRS scores of 4–6), we employed an unpaired 2-tailed t-test. Furthermore, temporal changes in the p75NTR/Creatine ratio were assessed using paired t-tests. Categorical demographic variables were analyzed with a Chi-squared test. We identified and excluded potential outliers from both cohorts where identified. This was achieved using the widely used ROUT method, with a false discovery rate set at Q = 1% [38]. All data analyses were conducted using SAS 9.4 (SAS Institute Inc., 2016, Cary, NC, USA) and Prism 9.4.1 (GraphPad Software, LLC, Boston, MA, USA). Descriptive figures were created with a licensed version of BioRender (Toronto, ON, Canada).

## 3. Results

### 3.1. Demographics and Disease Characteristics of Enrolled Patients

Forty-six urine samples were collected from three different groups of ABI patients at two time points: aSAH (*n* = 22, 47.8%), TBI (*n* = 8, 17.4%), and IS (*n* = 16, 34.8%) (Figure 1). In addition, we had four healthy control patients. The age distribution across the entire patient cohort ranged from 23 to 75 years. The mean age for ABI patients was 57.8 ± 13.3 years, while for the healthy cohort it was 44.00 ± 16.95 years. Different outcome groups as well as ABI patients and healthy controls were matched in terms of age and gender (Table 1 and Supplementary Table S2). Demographic analysis revealed a slightly higher proportion of male patients (26/46, 56.5%). The gender distribution among healthy control individuals was two males (50%) and two females (50%) (Table 1). Of the patients, 65.2% (30/46) of the cohort had a favorable outcome, while 34.8% (16/46) suffered from an unfavorable outcome (Table 1).

Out of the patients with favorable outcomes, the distribution of different brain injuries was 13 aSAH (43.3%), 3 TBI (10%), and 14 IS (46.7%) patients. Nine patients with unfavorable outcomes suffered from aSAH (56.3%), five patients from TBI (31.3%), and two patients from IS (12.5%) (Table 1). Patients suffering from aSAH included 22 patients in our cohort. Age varied from 34 to 75 years with the mean age being 55.8 ± 12.5 years in the favorable group and 58.9 ± 14.6 in the unfavorable group. In the outcome subgroups, there were six males (46.2%) and seven (53.8%) females and three males (33.3%) and six (66.7%) females, respectively (Supplementary Table S1). In the IS subcohort, there were 16 patients with an age range of 37 to 71 years. Mean age was 60.4 ± 11.4 years in the favorable group and 55.0 ± 21.2 in the unfavorable group. There were eight males (57.1%) and six females (42.9%) in the favorable group, and in the unfavorable group, both patients (*n* = 2) were males. In our TBI subcohort, we had eight patients. Age varied from 23 to 71 years with the mean age being 68.0 ± 23.3 years in the favorable group and 60.0 ± 12.3 years in the unfavorable group. There were seven (87.5%) males and one (12.5%) female in the group (Supplementary Table S1).

### 3.2. p75NTR in Urine after ABIs

**Figure 2:**
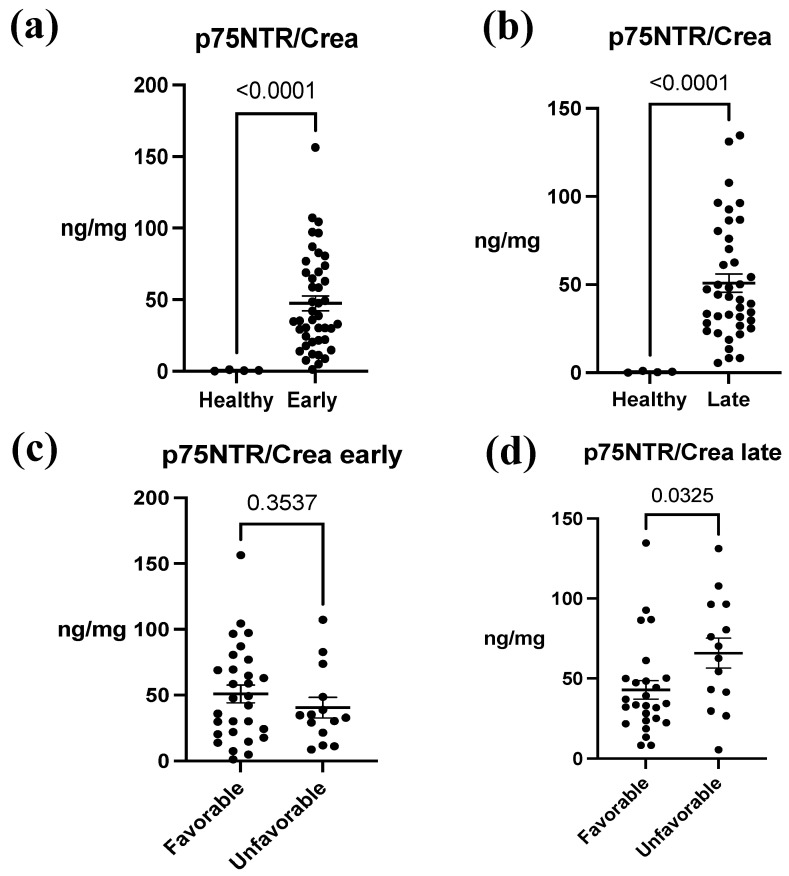
The association between urine p75NTR/Crea concentration ratio between acute brain injury (ABI) patients and healthy controls and between ABI outcomes. (**a**) A significant difference in p75NTR/Crea levels between early urine samples of ABI patients and healthy control patients was identified (*p* < 0.0001). (**b**) In addition, there was a significant difference in p75NTR/Crea levels from late urine samples of ABI patients and healthy control patients (*p* < 0.0001). (**c**) There was no significant association between p75NTR/Crea and mRS-measured outcome groups in early samples (*p* = 0.354). (**d**) In late samples, p75NTR/Crea concentration was significantly higher in patients with unfavorable outcomes (*p* = 0.033). Early = 1.50 ± 0.70 days; late = 9.17 ± 3.40 days. Favorable = modified Rankin Scale (mRS) 0–3; unfavorable outcome = mRS 4–6. The data represent mean ± SEM (p75NTR/Crea, ng/mg). Each individual black data point within a set corresponds to a single study patient.

**Figure 3:**
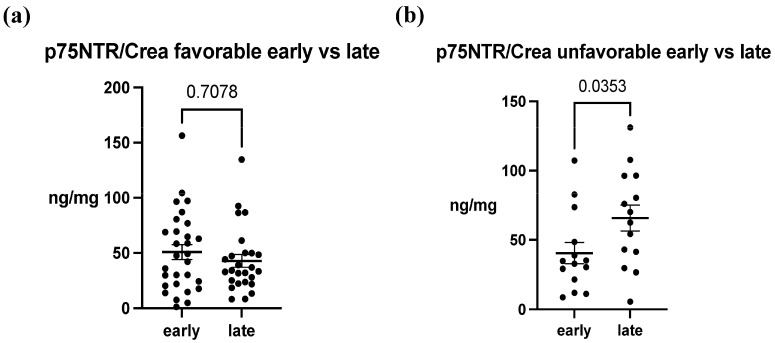
The change in p75NTR/Crea concentration ratio over time. (**a**) In the favorable outcome group, temporal difference in p75NTR/Crea was not observed (*p* = 0.708). (**b**) In the unfavorable group, p75NTR/Crea level was significantly increased in late samples (*p* = 0.0353). The data represent mean ± SEM (p75NTR/Crea, ng/mg). Each data point within a set corresponds to a single study patient.

In the analysis of p75NTR concentrations between ABI patients and healthy controls, a notable difference was observed between these groups. The mean concentration in healthy patients was 0.542 ± 0.440 ng/mg, whereas in ABI patients, the mean concentrations ranged from 40.49 ± 28.83 ng/mg to 65.85 ± 35.04 ng/mg. Overall, there was an approximately 100-fold increase in p75NTR concentration, and this difference was statistically significant in both early and late samples (*p* < 0.0001) (Figure 2a,b). Furthermore, in early urine samples, the difference in concentrations between the outcome groups was not statistically significant (favorable 50.86 ± 36.10 ng/mg, unfavorable 40.49 ± 28.83 ng/mg, *p* = 0.3537) (Figure 2c). In contrast, in the late urine samples, p75NTR concentration was significantly increased in the unfavorable group (favorable 42.79 ± 29.24 ng/mg, unfavorable 65.85 ± 35.04 ng/mg, *p* = 0.0325) (Figure 2d). When we examined the concentration differences between early and late samples between the groups, we noticed that in the favorable group, there was no significant (*p* = 0.9923) variation in p75NTR concentration over time (Figure 3a). Conversely, in the unfavorable group, there was a statistically significant increase in the concentration over time (*p* = 0.0353) (Figure 3b).

**Figure 4:**
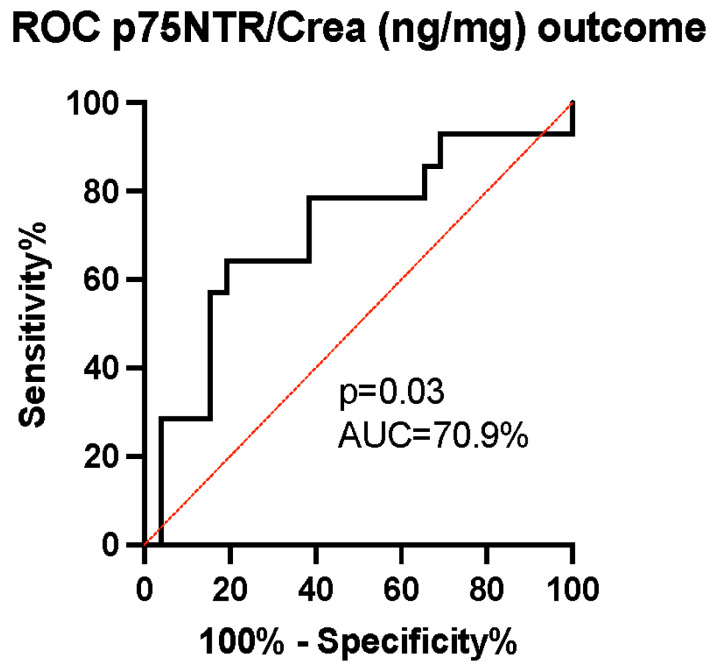
The receiver operating characteristic curve analysis of the p75NTR/Crea concentration ratio for prognostication of outcomes in late patient samples. We identified a strong discriminatory ability of p75NTR/Crea levels (ng/mg) in urine (area under the curve (AUC) 70.9%, (95% CI = 53–89%), *p* = 0.03), with an increased p75NTR/Crea ratio predicting unfavorable outcomes.

**Figure 5:**
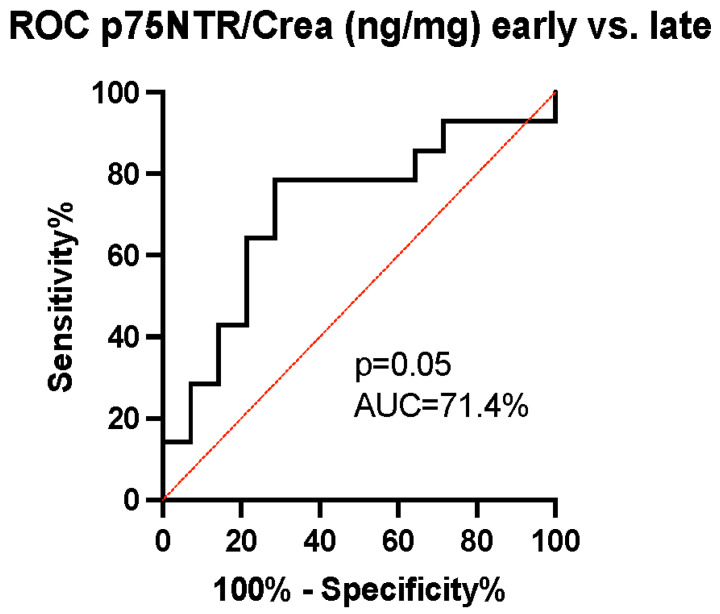
The receiver operating characteristic curve analysis of temporal changes in p75NTR/Crea concentration ratio. We identified a good discriminatory ability of p75NTR/Crea level (ng/mg) changes overtime in urine (area under the curve (AUC) 71.4%, (95% CI = 52–91%), *p* = 0.03). An increase in p75NTR/Crea ratio predicts unfavorable outcomes.

The ability of p75NTR concentration to predict patients’ mRS-measured outcomes was evaluated using the receiver operating characteristic (ROC) curve. The area under the curve (AUC) was calculated to be 70.9% with a 95% confidence interval (CI) of (53–89%) and a *p*-value of 0.03, indicating the good ability of p75NTR levels to predict dichotomized outcomes (Figure 4). As the p75NTR concentration was observed to increase over time between early and late samples in the unfavorable outcome group (Figure 3b), we conducted a further analysis using ROC curves to assess the ability of the change in p75NTR from early to late samples to predict the probability of an unfavorable outcome. The AUC was calculated to be 71.4% with a 95% CI of (52–91%) and a *p*-value of 0.05, suggesting that the temporal increase in p75NTR serves as an effective marker for predicting an unfavorable outcome (Figure 5).

In the favorable group, the mean p75NTR concentration decreased from 50.86 ± 36.10 ng/mg to 42.79 ± 29.24 ng/mg between the early and late timepoints, with a similar trend seen in the median concentration from 47.65 ng/mg to 33.84 ng/mg. Conversely, in the unfavorable group, the mean concentration increased from 40.49 ± 28.83 to 65.85 ± 35.04 ng/mg, and the median concentration increased from 33.80 ng/mg to 66.40 ng/mg (Table 1). There was a significantly higher p75NTR concentration in patients with unfavorable outcomes compared to those with favorable outcomes (*p* = 0.0325).

## 4. Discussion

In our study, we analyzed p75NTR concentrations in urine samples from patients with IS, TBI, and aSAH, to investigate their potential as biomarkers correlating with patient neurological outcomes. We found a distinct pattern where higher p75NTR levels were consistently associated with unfavorable outcomes at a 3-month follow-up, particularly in the later samples. Notably, early samples did not show significant differences in p75NTR levels between the different outcome groups, suggesting that the prognostic value of p75NTR becomes more pronounced over time. A critical observation was the significantly higher p75NTR levels in urine from ABI patients compared to healthy control subjects, a trend observed irrespective of the patients’ future outcomes. These findings underline the potential of urinary p75NTR not only as a prognostic indicator for patient outcomes in ABIs but also as a promising diagnostic biomarker across various types of brain injuries.

Our findings of elevated urinary p75NTR concentrations in patients with unfavorable outcomes after ABI align with existing literature that reports elevations in various chronic neurodegenerative diseases. This correlation not only reinforces the established role of p75NTR in neuronal damage but also extends its relevance to acute contexts like IS, TBI, and aSAH. The overexpression of p75NTR observed in animal models following ABIs and its association with stroke-induced sensorimotor deficits in preclinical studies further corroborate our results [22,31,39]. Our study adds to this evidence by demonstrating a clear temporal increase in p75NTR levels, particularly in patients with unfavorable outcomes, suggesting that these elevated levels might originate directly from the damaged brain tissue.

The distinct pattern of p75NTR concentrations we observed, especially the significant increase over time in the unfavorable outcome group, which was not observed in patients with favorable outcomes, offers new insights into the dynamic nature of p75NTR post-injury. This temporal alteration underscores the potential of p75NTR as a biomarker that not only indicates the presence of neuronal damage but also provides prognostic information about the severity and progression of the diseases. Our study thus bridges the gap between preclinical findings and clinical application, suggesting that urinary p75NTR could be a viable and informative biomarker for assessing patient outcomes after ABIs.

In previous studies within the field, urinary p75NTR concentrations in healthy control patients was consistent with the control patients in our investigation [32,34]. This underpins the reliability of our measurement techniques and novelty of ABI-related findings. Nonetheless, our study revealed significantly elevated concentrations in patients with ABIs in contrast to these controls, approximately 100-fold. In ALS patients, the concentration of p75NTR rose to approximately three times that of healthy individuals [34]. This finding supports the hypothesis that acute brain injuries can substantially elevate the concentration of p75NTR detected in urine, suggesting its potential as a diagnostic marker. In neurodegenerative diseases, the concentrations of p75NTR may be comparatively lower than in acute brain injuries, possibly due to the lesser extent of acute neuronal damage [40]. As a marked increase in p75NTR may be indicative of the presence of a brain injury, the identified upward trend persisting over time may impart prognostic value.

**Figure 6:**
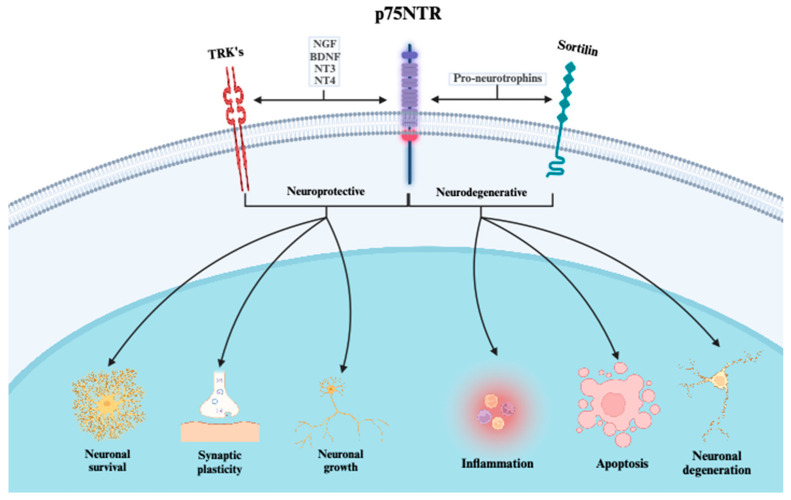
The distinct signaling mechanisms of the p75 neurotrophin receptor and its co-receptors. When p75NTR interacts with TRK-receptors (A, B, C), mature neurotrophins (NGF, BDNF, NT3, and NT4) serve as ligands. The effects of these interactions are primarily neuroprotective. However, when interacting with sortilin, the affinity for pro-neurotrophins increases, leading primarily to neurodegenerative effects. p75NTR = p75 neurotrophin receptor; TRK = receptor tyrosine kinase; NGF = neuro growth factor; BDNF = brain-derived neurotrophic factor; NT3 = neurotrophin-3; NT4 = neurotrophin-4.

One important finding from this study is the clear temporal relationship between ABI and elevated p75NTR levels. This finding can partly be explained by the molecular characteristics of p75NTR [15]. Under normal physiological conditions, p75NTR is downregulated, whereas upregulation is initiated following neuronal stress [22,41,42] (Figure 6).

The observed increase in urinary p75NTR concentrations aligns with the known pattern of delayed expression response. Our findings, showing elevated levels of p75NTR in later urine samples, corroborate this understanding. This delayed pattern may be explained by the molecular interactions of p75NTR, particularly its binding with pro-apoptotic factors like proNGF and sortilin [18,20]. Upon such interactions, the extracellular component of p75NTR is believed to be cleaved and subsequently excreted into the urine, accounting for the higher concentrations observed in later samples [34]. Consequently, this mechanism leads to a temporal disparity in p75NTR levels, with no significant differences in early samples but a marked increase in later samples. This temporal increase, especially in patients with unfavorable outcomes, underscores the potential of p75NTR as a reflective biomarker of ongoing neuronal processes post-injury. Additionally, considering the complex dual role p75NTR has towards neurons, capable of both neuronal constructive and destructive functions [15,16], it could be possible that the concentration remains elevated for an extended period after the injury.

An additional mechanism related to neurotrophin signaling is that BDNF overexpression modulates intracellular calcium levels, which offers neuroprotection against ischemic conditions and excitotoxicity [43,44]. This regulation of calcium homeostasis appears crucial in preventing the overload of Ca^2+^ that triggers apoptotic and necrotic processes in neurons [43,44]. Particularly notable is the alteration in the expression and function of NMDA and AMPA receptor subunits, which modulates calcium conductivity, further illustrating the intricate relationship between BDNF activity and calcium dynamics [43]. In addition, effects similar to BDNF may also arise from the direct inhibition of L-type calcium channels, which in turn activate TrkB receptor signaling [45]. This highlights an additional layer of complexity where calcium signaling intersects with neurotrophic factors, offering additional perspective on the potential therapeutic approaches for brain injury.

The therapeutic modulation of p75NTR has gained attention in the neurological research field, particularly evidenced by studies where blocking p75NTR with pharmacological agents like LM11A-31 and EVT901 led to improved outcomes in brain injury animal models [25,31]. Reflecting these findings, our study observed a significant increase in p75NTR concentration at later stages post-injury, which aligns with the temporal aspect of these therapeutic interventions. This finding underscores the prognostic value of p75NTR as a function of time, suggesting its applicability not only in understanding the injury’s progression but also in evaluating the response to specific treatments. Consequently, our results highlight the potential of urinary p75NTR to serve as an invaluable monitoring biomarker offering a novel non-invasive approach to assess the effectiveness of pharmacological interventions in future clinical trials.

The choice of urine as a biofluid for biomarker analysis offers several practical advantages that are particularly relevant in the clinical setting. Its easy accessibility stands out as a major benefit, eliminating the need for more invasive procedures typically associated with blood sampling. This aspect of urine collection not only enhances patient comfort but also may reduce procedural risks. Additionally, the non-invasive nature of urine sampling is conducive to repeated and large-volume collections, allowing for more comprehensive and longitudinal monitoring of biomarker levels, a crucial factor in the dynamic nature of ABIs. Furthermore, the stability of urine proteins at room temperature, as evidenced by numerous studies, adds to the utility of urine in clinical applications [33,46,47]. This stability ensures that the integrity of the biomarkers, including p75NTR, is maintained, facilitating accurate analysis even when immediate processing is not feasible. This aspect of urine analysis is particularly advantageous in settings where advanced storage facilities may not be readily available. The combined benefits of accessibility, non-invasiveness, and stability make urine an ideal medium for biomarker research, offering a practical and efficient approach to the early detection and ongoing monitoring of conditions like ABIs.

## 5. Limitations

Our study acknowledges several limitations that warrant consideration in the interpretation of our findings. Firstly, the disparity in the distribution of favorable and unfavorable outcome patients within our sample may introduce bias in the comparative analysis. A more balanced, or propensity matched, distribution would facilitate a more nuanced understanding of the relationships observed.

Secondly, the modest sample size of 46 participants potentially constrains the generalizability and statistical power of our findings. Although we took measures to ameliorate this by analyzing urinary p75NTR concentrations at two distinct time points post-admission, the validity of our results would be bolstered by replicating this study with a larger, multicentric cohort. In addition, the number of our control patients (*n* = 4) was small; however, the results were in line with previously presented studies.

Thirdly, the inclusion of three distinct types of acute brain injuries (aSAH, IS, and TBI) adds a layer of complexity due to the inherent heterogeneity in disease pathologies and patient responses. Our cohort exhibits significant variation in the location of brain injury, which, in turn, complicates the comparison of patient outcomes. While our results suggest that p75NTR concentrations may serve as a common prognostic marker irrespective of injury type, a more exhaustive exploration across individual injury types may yield deeper insights into the specific roles and implications of p75NTR. In addition, while the use of commercial ELISA plates provided specific and sensitive measurement of p75NTR levels, we acknowledge that relying solely on one technique limits the scope of our analysis. Future studies would benefit from incorporating additional methods such as mass spectrometry offering a more comprehensive validation and exploration of these findings.

Lastly, the multifaceted nature of acute brain injury outcomes necessitates a broad approach. Future investigations could enhance the robustness of the findings by examining an array of other potential biomarkers. Incorporating these aspects, for example, into an artificial intelligence platform would contribute to a more comprehensive understanding of the intricate mechanisms influencing patient outcomes following acute brain injuries.

In conclusion, while our study provides preliminary evidence of the prognostic utility of easily accessible urinary p75NTR concentrations in different acute brain injuries, addressing these limitations in future research would significantly strengthen the evidence and contribute to the evolving landscape of neurobiomarker discovery and validation.

## 6. Conclusions

In conclusion, our study highlights that an increase in urinary p75NTR concentration is intricately linked with patient outcomes in a temporal manner across various types of ABIs. We have established that p75NTR functions effectively as a prognostic marker, irrespective of the specific type of ABI. Notably, patients with ABI exhibit significantly higher p75NTR concentrations in their urine compared to healthy controls, supporting the hypothesis that p75NTR could be a viable diagnostic biomarker for ABIs. Ultimately, our research opens new avenues for the development of diagnostic, prognostic, and monitoring biomarkers.

## Figures and Tables

**Table 1:** Characteristics and concentrations of urine p75NTR/Crea from the acute brain injury cohort (*n* = 46) and healthy individuals using a modified Rankin Scale (mRS). Favorable mRS 0–3; unfavorable mRS 4–6.

Variables	Favorable (*n* = 30)	Unfavorable (*n* = 16)	*p*-Value
Age in years			0.7283
Mean ± SD	57.3 ± 13.3	58.8 ± 13.7	
Min–Max	23.0–75.0	34.0–74.0	
Median (IQR)	60.0 (48.0–68.0)	65.0 (45.0–70.0)	
Sex			0.9783
Male	17 (56.7)	9 (56.3)	
Female	13 (43.3)	7 (43.8)	
Type of brain injury			0.0373
aSAH	13 (43.3)	9 (56.3)	
TBI	3 (10.0)	5 (31.3)	
IS	14 (46.7)	2 (12.5)	
p75NTR/Crea Early (ng/mg)			0.3537
Mean ± SD	50.86 ± 36.10	40.49 ± 28.83	
Min–Max	1.3–156.4	8.7–107.3	
Median (IQR)	47.65 (21.3–73.2)	33.80 (19.2–54.8)	
p75NTR/Crea Late (ng/mg)			0.0325
Mean ± SD	42.79 ± 29.24	65.85 ± 35.04	
Min–Max	8.3–134.7	5.6–131.2	
Median (IQR)	33.84 (23.4–50.0)	66.40 (38.5–96.4)	
p75NTR/Crea Healthy (ng/mg)	Healthy (*n* = 4)		<0.0001 †
Mean ± SD	0.542 ± 0.440 0.084–1.124 0.479 (0.155–0.991)		
Min–Max			
Median (IQR)			
Age in years			>0.05 ‡
Mean ± SD	44.00 ± 16.95		
Min–Max	33.0–69.0		
Median (IQR)	37.0 (33.3–62.8)		
Sex			>0.05 ‡
Male	2 (50.0)		
Female	2 (50.0)		

† Statistical comparisons of p75NTR/Crea between healthy versus favorable, and healthy versus unfavorable outcome groups (both comparisons *p* < 0.0001). ‡ Statistical comparisons of age and sex between healthy versus favorable, and healthy versus unfavorable outcome groups (both comparisons *p* > 0.05).

## Data Availability

The anonymized data from this study can be made available upon request to qualified researchers who have obtained appropriate institutional review board (IRB) approval. Requests should be directed to the corresponding author (J.K.).

## Supplementary Materials

The following supporting information can be downloaded at: https://www.mdpi.com/article/10.3390/biomedicines12010112/s1, Refs. [33,48,49]. Figure S1: The association of p75NTR/Crea concentration and outcome in Acute Brain Injuries. Disease-specific analysis. (a–c) There was no significant association between p75/Crea and mRS-measured outcome groups in early samples of aSAH, IS and TBI subgroups (*p* = 0.2370, *p* = 0.5756 and *p* = 0.9345 respectively). (d–f) In late samples, p75/Crea concentration showed rising trend for patients with unfavorable outcome in all three sub-groups. In IS group the association between concentration and mRS-measured outcome was significant (*p* = 0.0004). In aSAH and TBI groups there was no significant association (*p* = 0.4512 and *p* = 0.7673 respectively). (g–i) Temporal change in concentration between early and late samples in aSAH, TBI and IS patients with favorable outcome showed no significant difference (*p* = 0.9594, *p* = 0.2500 and *p* = 0.0506 respectively). (j–l) Temporal change in concentration between early and late samples in aSAH, TBI and IS patient with unfavorable outcome showed no significant difference (*p* = 0.0547, *p* = 0.3125, *p* = n/a respectively). However, the trend was rising over time in all three subgroups; Table S1: Demographic and disease characteristics of the studied cohort; Table S2: Characteristics of combined cohort.
