# Supplementary material for: Brain Plasticity Modulator p75 Neurotrophin Receptor in Human Urine after Different Acute Brain Injuries—A Prospective Cohort Study
Source: Biomedicines. 2024 Jan 5;12(1):112. doi: 10.3390/biomedicines12010112 (PMC10813252; doi:10.3390/biomedicines12010112)
Supplement: Supplementary file 1 [file biomedicines-12-00112-s001.zip › Supplemental Table S1.pdf]

Supplemental Table S1. Demographic and disease characteristics of the studied cohort.

Aneurysmal subarachnoid hemorrhage

	Favorable (mRS 0-3, n=13)	Unfavorable (mRS 4-6, n=9)	p-value
**Age in years**			0.5968
Mean±SD	55.8±12.5	58.9±14.6	
Min–Max	38.0–75.0	34.0–74.0	
Median (IQR)	59.0 (43.0–65.0)	65.0 (47.0–70.0)	
**Sex**			0.5476
Male	6 (46.2)	3 (33.3)	
Female	7 (53.8)	6 (66.7)	
**GCS scene**			0.1200
Mean±SD	12.8±3.7	9.7±5.5	
Min–Max	5.0–15.0	3.0–15.0	
Median (IQR)	15.0 (13.0–15.0)	12.0 (4.0–15.0)	
**Neuroworsening**			0.0180
No	8 (61.5)	1 (11.1)	
Yes	5 (38.5)	8 (88.9)	
**Modified Fisher Scale**			0.3325
Mean±SD	3.5±0.8	3.8±0.7	
Min–Max	2.0–4.0	2.0–4.0	
Median (IQR)	4.0 (3.0–4.0)	4.0 (4.0–4.0)	
**H&H**			0.0205
Mean±SD	2.3±1.4	3.8±1.3	
Min–Max	1.0–5.0	2.0–5.0	
Median (IQR)	2.0 (1.0–3.0)	4.0 (3.0–5.0)	
**Location of Aneurysm**			0.2507
ACA	1 (7.7)		
AcomA	5 (38.5)	3 (33.3)	
MCA	1 (7.7)	3 (33.3)	
BAS	1 (7.7)		
PcomA		2 (22.2)	
PICA	1 (7.7)		
ICA	4 (30.8)	1 (11.1)	
**Diagnosis of Acute Hydrocephalus**			0.5121
No	9 (69.2)	5 (55.6)	
Yes	4 (30.8)	4 (44.4)	
**Clinical DCI**			0.1324
No	11 (84.6)	5 (55.6)	
Yes	2 (15.4)	4 (44.4)	

Ischemic stroke

	Favorable (mRS 0-3, n=14)	Unfavorable (mRS 4-6, n=2)	p-value
**Age in years**			0.5703
Mean±SD	60.4±11.4	55.0±21.2	
Min–Max	37.0–74.0	40.0–70.0	
Median (IQR)	64.0 (51.0–71.0)	55.0 (40.0–70.0)	
**Sex**			0.2416
Male	8 (57.1)	2 (100)	
Female	6 (42.9)		
**Neuroworsening**			0.4677
No	11 (78.6)	2 (100)	
Yes	3 (21.4)		
**Type of infarction**			0.2301
Cardiogenic	4 (28.6)		
Thrombosis	5 (35.7)		
Cryptic	5 (35.7)	2 (100)	
**Volume of infarction (ml)**			0.5488
Mean±SD	10.3±16.0	58.5±79.6	
Min–Max	0.0–46.8	2.2–114.8	
Median (IQR)	3.7 (0.4–6.4)	58.5 (2.2–114.8)	

Traumatic brain injury

	Favorable (mRS 0-3, n=3)	Unfavorable (mRS 4-6, n=5)	p-value
**Age in years**			0.4295
Mean±SD	68.0±24.3	60.0±12.3	
Min–Max	23.0–71.0	42.0–71.0	
Median (IQR)	54.0 (23.0–71.0)	65.0 (47.5–70.0)	
**Sex**			0.4816
Male	3 (100)	4 (80)	
Female	0 (0)	1 (20)	
**Neuroworsening**			0.7246
No	1 (33.3)	1 (20)	
Yes	2 (66.6)	4 (80)	
**Thickness of aSDH (mm)**			0.2805
Mean±SD	12.67±7.5	8.1±3.68	
Min-Max	5.0–20.0	4.5–12.0	
Meadian (IQR)	13.0 (5.0–20.0)	7.0 (4.75–12.0)	
**Midline shift (mm)**			0.5008
Mean±SD	8.33±7.64	5.6±3.4	
Min–Max	0.0–15.0	0.0–9.0	

Median (IQR)	10.0 (0.0–15.0)	7.0 (2.5–8.0)

mRS=Modified Ranking Scale, H&H=Hunt and Hess Classification
ACA=Anterior Cerebral Artery, AcomA=Anterior Communicans Artery, MCA=Middle Cerebral Artery, BAS=Basilar Artery, PICA=Posterior Inferior Cerebellar Artery, ICA=Internal Carotic Artery
DCI=Delayed Clinical Ischemia, GCS=Glasgow Coma Scale
