# Supplementary material for: Brain Plasticity Modulator p75 Neurotrophin Receptor in Human Urine after Different Acute Brain Injuries—A Prospective Cohort Study
Source: Biomedicines. 2024 Jan 5;12(1):112. doi: 10.3390/biomedicines12010112 (PMC10813252; doi:10.3390/biomedicines12010112)
Supplement: Supplementary file 1 [file biomedicines-12-00112-s001.zip › Supplemental Table S2.pdf]

Supplemental Table S2. Characteristics of combined cohort.

Variables	Statistics (n=46)
**Age in years**	
Mean±SD	57.8±13.3
Min–Max	23.0–75.0
Median (IQR)	62.0 (47.0–70.0)
**Sex**	
Male	26 (56.5)
Female	20 (43.5)
**Type of brain injury**	
Aneurysmal subarachnoid hemorrhage	22 (47.8)
Traumatic brain injury	8 (17.4)
Ischemic stroke	16 (34.8)
**mRS**	
Favorable (mRS=0-3)	30 (65.2)
Unfavorable (mRS=4-6)	16 (34.8)
