# Supplementary material for: Brain Plasticity Modulator p75 Neurotrophin Receptor in Human Urine after Different Acute Brain Injuries—A Prospective Cohort Study
Source: Biomedicines. 2024 Jan 5;12(1):112. doi: 10.3390/biomedicines12010112 (PMC10813252; doi:10.3390/biomedicines12010112)
Supplement: Supplementary file 1 [file biomedicines-12-00112-s001.zip › Supplementary material.pdf]

Brain Plasticity Modulator p75 Neurotrophin Receptor in Human Urine after Different Acute Brain Injuries – A Prospective Cohort Study

Santtu Hellström[1,2], Antti Sajanti[1], Abhinav Srinath[3], Carolyn Bennett[3], Romuald Girard[3], Ying Cao[4], Janek Frantzén[1], Fredrika Koskimäki[5], Johannes Falter[6], Seán B. Lyne[7], Tomi Rantamäki[8,9], Riikka Takala[10], Jussi P. Posti[1], Susanna Roine[5], Jukka Puolitaival[11], Miro Jänkälä[11], Sulo Kolehmainen[12], Melissa Rahi[1], Jaakko Rinne[1], Eero Castrén[12], Janne Koskimäki[1,11,12]

[1]Division of Clinical Neurosciences, Department of Neurosurgery, Turku University Hospital and University of Turku, P.O. Box 52, Hämeentie 11, FI-20521, Turku, Finland.

[2]Faculty of Medicine and Health Technology, Tampere University, 33520 Tampere, Finland.

[3]Neurovascular Surgery Program, Section of Neurosurgery, The University of Chicago Medicine and Biological Sciences, 5841 S. Maryland, Chicago, IL 60637, US.

[4]Department of Radiation Oncology, Kansas University Medical Center, Kansas City, KS 66160, USA

[5]Neurocenter, Acute Stroke Unit, Turku University Hospital, P.O. Box 52, FI-20521, Turku, Finland

[6]Department of Neurosurgery, University Medical Center of Regensburg, Regensburg, Germany

[7]Department of Neurosurgery, Brigham and Women's Hospital, Harvard Medical School, Boston, Massachusetts.

[8]Laboratory of Neurotherapeutics, Molecular and Integrative Biosciences Research Programme, Faculty of Biological and Environmental Sciences and Drug Research Program

[9]Division of Pharmacology and Pharmacotherapy, Faculty of Pharmacy, University of Helsinki, Finland

[10]Perioperative Services, Intensive Care and Pain Medicine, Turku University Hospital and Univeristy of Turku, POB 52, 20521, Turku, Finland.

[11]Department of Neurosurgery, Oulu University Hospital, Box 25, 90029 OYS, Finland.

[12]Neuroscience Center, HiLIFE, University of Helsinki, Box 63, 00014 Helsinki, Finland.

Corresponding author:

Janne Koskimäki, MD, PhD, Associate Professor

Neurocenter, Department of Neurosurgery, Turku University Hospital and University of Turku, P.O. Box 52 (Hämeentie 11), FI-20521, Turku, Finland.

E-mail: koskimaki@uchicago.edu

Supplementary results:

Demographics and disease characteristics of enrolled patients

A breakdown of our cohort according to disease groups is presented in **Table S1**, detailing specific clinical characteristics associated with each subgroup. In the aneurysmal subarachnoid hemorrhage (aSAH) cohort, significantly higher Hunt and Hess (H&H) scores were observed in patients with an unfavorable outcome (p=0.0205), validating an association between the severity of hemorrhage and patient prognosis (**Suppl. Table S1**). Out of the favorable outcome group, 4 patients (30.8%) were diagnosed with acute hydrocephalus, while 4 patients (44.4%) in the unfavorable outcome group experienced this condition. However, the observed difference was not statistically significant (p=0.5121), indicating that the incidence of acute hydrocephalus did not significantly impact the overall outcomes (**Suppl. Table S1**). Patients included in favorable group was found a wider range of aneurysm locations, with a total of 6 different locations. The most prevalent locations were the anterior communicating artery (AcomA) (38.5%) and internal carotid artery (ICA) (30.8%). Unfavorable group presented 4 different aneurysm locations in our cohort. The most prevalent locations were AcomA (33.3%) and middle cerebral artery (MCA) (33.3%) (**Suppl. Table S1**).

In the ischemic stroke (IS) subcohort we analyzed the type of the infarction and infarction volume. In the favorable outcome subgroup of IS cases, the distribution of infarction types included: four cardiogenic (28.6%), five thrombosis 35.7%), and five cryptogenic (35.7%). Within the unfavorable outcome subgroup, cryptogenic infarction was observed in both cases (100%). There was clear numerical difference between the outcome groups, but statistical analysis did not demonstrate significant differences in the volumes of infarction between the subgroups (p=0.5488). This can be explained by the low number of unfavorable IS patients in the cohort and with the fact that in that group there was one small and one large infarct. (n=2) (**Suppl. Table S1**).

We completed further analysis for the traumatic brain injury (TBI) sub-cohort and measuring thickness of acute subdural hematoma (aSDH) and midline shift. For cases resulting in a favorable outcome, the mean aSDH thickness measured 12.67 ± 7.5 mm, with a median of 13.0 mm. Cases with an unfavorable

outcome exhibited a smaller mean thickness of 8.1 ± 3.68 mm and a median of 7.0 mm. This difference in aSDH thickness was not statistically significant (p=0.2805) (**Suppl. Table S1**). Among cases with a favorable outcome, the mean midline shift was 8.33 ± 7.64 mm, with a median of 10.0 mm. In cases resulting in an unfavorable outcome, the mean midline shift was slightly smaller at 5.6 ± 3.4 mm, with a median of 7.0 mm. Like aSDH thickness, the differences observed in midline shift did not achieve statistical significance (p=0.5008) (**Suppl. Table S1**).

p75NTR concentration in disease-specific groups

In our additional concentration analyses, we investigated the disease-specific impact of p75NTR/Crea ratio on patient outcome, both in early and late samples, as well as with the specific outcome. In the early samples of aSAH patients, no significant differences were observed in relation to p75NTR/Crea ratio and patient outcomes (p=0.237) (**Suppl. Figure S1a**). Similarly, in the early samples of TBI and IS groups, no significant differences were noted (p=0.5756 and p=0.9345, respectively) (**Suppl. Figure S1b-c**). In the late samples of the aSAH group, the p75NTR/Crea ratio was higher in the unfavorable outcome subgroup, but this result was not statistically significant (p=0.4512) (**Suppl. Figure S1d**). In the TBI group, the ratio was slightly higher in the unfavorable outcome subgroup, but again, the result was not statistically significant (p=0.7673) (**Suppl. Figure S1e**). Notably, the IS group exhibited significantly higher concentrations in the unfavorable outcome subgroup (p=0.0004) (**Suppl. Figure S1f**). Overall, clear trends towards higher ratios in late samples were observed in unfavorable outcome patients.

Temporal changes of p75NTR concentration in disease-specific groups

We also examined disease groups based on outcomes and compared the concentration differences between early and late samples, essentially assessing changes in p75NTR/Crea concentration over time.

When examining the favorable outcome group, we observed that in the aSAH group, the concentration remained relatively stable between early and late samples, and this result was not statistically significant (p=0.9594) (**Suppl. Figure S1g**). In the TBI group, there was a higher concentration in the late samples, but this difference was not statistically significant (p=0.25) (**Suppl. Figure S1h**). In the IS group, we

observed a decrease in p75NTR/Crea concentration, and this result was nearly statistically significant (p=0.0506) (**Suppl. Figure S1i**). When we examined the unfavorable outcome group, in each disease group, there was an increasing trend in concentration over time. In the late samples, there were higher p75NTR/Crea levels in both the aSAH, TBI, and IS groups (p=0.0547, p=0.3125, and p=not applicable, respectively). A clear temporal trend emerged in the unfavorable group (**Suppl. Figure S1j-l**).

Supplementary discussion

When we analyzed the association between p75NTR concentration and patients' outcome in disease specific subgroups (aSAH, IS and TBI), we found no significant differences in early samples. On the other hand, late samples displayed significantly higher p75NTR concentration in patients with IS and clear temporally increasing trends in patients with aSAH and TBI were observed.

Patients experiencing an unfavorable outcome exhibited elevated p75NTR concentrations in late samples across all disease groups. Although these results did not reach statistical significance, they bolster our primary findings, suggesting a link between p75NTR levels and poorer neuronal outcomes in patients. Our results reveal a clear temporal upward trend in p75NTR concentration between early and late samples, with the p75NTR concentration being higher in the unfavorable outcome group. Similar findings of a directly proportional association between urinary p75NTR and severity neurological disease have been observed in conditions such as ALS(1). In addition, trials revealed a late increase in p75NTR concentration when comparing plasma levels between TBI patients and healthy individuals, with the peak values occurring 10 days post-TBI(2). Furthermore, in patients with mechanical trauma, the p75NTR concentration exhibited a progressively increasing trend over time, showing a significant correlation with patient outcome(3). These findings support our hypothesis regarding the potential role of p75NTR as a prognostic marker in acute brain injuries and are consistent with our observation of the delayed elevation of p75NTR following the injury.

References

1. Shepheard SR, Wuu J, Cardoso M, Wiklendt L, Dinning PG, Chataway T, et al. Urinary p75ECD: A prognostic, disease progression, and pharmacodynamic biomarker in ALS. Neurology. 2017 Mar 21;88(12):1137–43.

2. Maier B, Lehnert M, Laurer HL, Mautes AE, Steudel WI, Marzi I. Delayed elevation of soluble tumor necrosis factor receptors p75 and p55 in cerebrospinal fluid and plasma after traumatic brain injury. Shock Augusta Ga. 2006 Aug;26(2):122–7.

3. Ertel W, Keel M, Bonaccio M, Steckholzer U, Gallati H, Kenney JS, et al. Release of anti-inflammatory mediators after mechanical trauma correlates with severity of injury and clinical outcome. J Trauma. 1995 Nov;39(5):879–85; discussion 885-887.

Supplemental Figure S1. The association of p75NTR/Crea concentration and outcome in Acute Brain Injuries. Disease-specific analysis. a-c) There was no significant association between p75/Crea and mRS-measured outcome groups in early samples of aSAH, IS and TBI subgroups (p=0.2370, p=0.5756 and p=0.9345 respectively). d-f) In late samples, p75/Crea concentration showed rising trend for patients with unfavorable outcome in all three sub-groups. In IS group the association between concentration and mRS-measured outcome was significant (p=0.0004). In aSAH and TBI groups there was no significant association (p=0.4512 and p=0.7673 respectively). g-i) Temporal change in concentration between early and late samples in aSAH, TBI and IS patients with favorable outcome showed no significant difference (p=0.9594, p=0.2500 and p=0.0506 respectively). j-l) Temporal change in concentration between early and late samples in aSAH, TBI and IS patient with unfavorable outcome showed no significant difference (p=0.0547, p=0.3125, p=n/a respectively). However, the trend was rising over time in all three subgroups.
