# Supplementary figures and images for: Brain Plasticity Modulator p75 Neurotrophin Receptor in Human Urine after Different Acute Brain Injuries—A Prospective Cohort Study
Source: Biomedicines. 2024 Jan 5;12(1):112. doi: 10.3390/biomedicines12010112 (PMC10813252; doi:10.3390/biomedicines12010112)

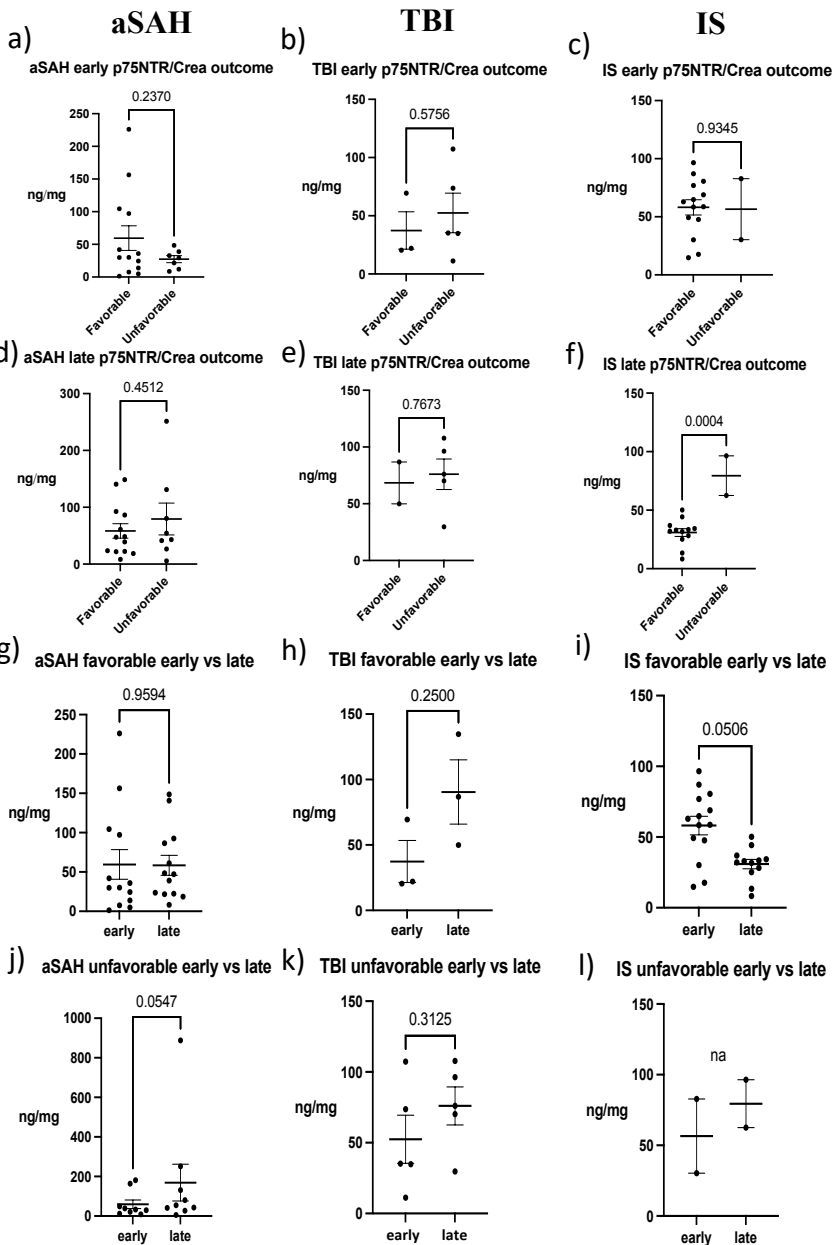

Supplement: Supplementary file 1 [file biomedicines-12-00112-s001.zip › Figure S1.pdf]
